# An Extension of Reversible Image Enhancement Processing for Saturation and Brightness Contrast

**DOI:** 10.3390/jimaging8020027

**Published:** 2022-01-28

**Authors:** Yuki Sugimoto, Shoko Imaizumi

**Affiliations:** 1 Graduate School of Science and Engineering, Chiba University, 1-33 Yayoicho, Inage-ku, Chiba 263-8522, Japan; yuki.3115-0423-1998@chiba-u.jp; 2 Graduate School of Engineering, Chiba University, 1-33 Yayoicho, Inage-ku, Chiba 263-8522, Japan

**Keywords:** contrast enhancement, saturation improvement, reversibility, data hiding, HSV color space

## Abstract

This paper proposes a reversible image processing method for color images that can independently improve saturation and enhance brightness contrast. Image processing techniques have been popularly used to obtain desired images. The existing techniques generally do not consider reversibility. Recently, many reversible image processing methods have been widely researched. Most of the previous studies have investigated reversible contrast enhancement for grayscale images based on data hiding techniques. When these techniques are simply applied to color images, hue distortion occurs. Several efficient methods have been studied for color images, but they could not guarantee complete reversibility. We previously proposed a new method that reversibly controls not only the brightness contrast, but also saturation. However, this method cannot fully control them independently. To tackle this issue, we extend our previous work without losing its advantages. The proposed method uses the HSV cone model, while our previous method uses the HSV cylinder model. The experimental results demonstrate that our method flexibly controls saturation and brightness contrast reversibly and independently.

## 1. Introduction

Currently, image processing applications are widely used to edit images. Most image processing methods, however, cannot consider reversibility; that is, the original images can never be retrieved from the output images. If a user desires to reconstruct an original image, the original image itself or editing information must be stored aside from the output image. This problem noticeably affects devices with limited storage capacity, such as tablets and smartphones. Therefore, it is desired to restore the original images from the output images without increasing the data amount. Reversible contrast enhancement (CE) [1,2,3,4,5,6,7,8,9,10,11,12,13,14,15] using reversible data hiding (RDH) is a technique where the brightness contrast can be flexibly enhanced and reverted without increases in data.

Data hiding is generally researched to prevent unauthorized use of images and detect tampering in images [16,17]. This technique is classified into two types: irreversible and reversible. The former has a high hiding capacity and high resistance against attacks, but the original images can never be retrieved after data extraction. In contrast, the latter has a relatively low hiding capacity and low resistance against attacks, but it can retrieve the original images after data extraction. The above reversible CE methods use the latter and aim for perfect reversibility by embedding additional information.

Most reversible CE methods [1,2,3,4,5,6,7,8,9,10,11,12] have been studied for grayscale images. If we simply use those methods for color images, some image distortion can appear in the output images. In contrast, some CE methods [13,14] have been proposed for color images. The images restored with these methods have a high image quality, but they do not perfectly match their originals.

Wu et al. [1] proposed a reversible CE method with a histogram-shifting (HS)-based RDH for grayscale images. This method accomplishes perfect reversibility by embedding additional information into image histograms to recover the original images. On the basis of Wu et al.’s method, many extended methods have been proposed. These methods aim to improve the hiding capacity, CE effect, and/or output image quality. Gao et al. [2] proposed a high-capacity method that embeds information into not only the space domain, but also the integer wavelet transform domain. Chen et al. [3] focused on the histogram distribution characteristics for effective CE. Their method also improves the image quality by limiting the range of pixel value modification due to HS. The HS process [1] was developed by Kim et al. [4,5] to improve the CE effect. Their method flexibly defines the direction in which an image histogram is shifted depending on the positions of the peak and minimum points in the histogram. Mansouri et al. [6] further extended the previous methods [4,5]. This method conducts CE by using the two highest bins simultaneously and achieves both high capacity and brightness preservation. Wu et al. [7] adopted two-dimensional image histograms. In this method, the CE effect is incremented, and image distortion is alleviated. The preprocessing of HS [1] was modified by Wu et al. [8]; consequently, the distortion caused by the preprocessing was effectively inhibited. For medical images, multiple CE methods have been studied [9,10,11,12]. These methods first divide an original image into regions of interest (ROIs) and non-regions of interest before the HS process; the CE effect thus appears in the ROIs only. The above methods are applicable only to grayscale images. Therefore, hue distortion is caused when we simply apply these methods to color images.

Wu et al. [13] proposed a reversible CE method for color images that uses the HSV color space. This method can enhance the brightness contrast, which is simply referred to as contrast, without saturation and hue distortion. Meanwhile, rounding errors are caused by preservation processes for saturation and hue. Therefore, although the retrieved images are high in quality, this method does not reconstruct the original images. Wu et al. [14] extended the method [13] to ensure perfect reversibility. The extended method enhances the contrast without hue distortion, but fails to preserve saturation. Additionally, in the recovery process, the R, G, and B values in some pixels might be mixed up in a restored image. In such a case, those pixels are not reverted to their originals. Consequently, this method still cannot ensure perfect reversibility, in common with the previous method [13].

To tackle perfect reversibility, we previously proposed another reversible image processing method for color images [15]. Our previous method embeds additional information by using the prediction error expansion with histogram shifting (PEE-HS) method [18]. Additionally, the method not only enhances contrast, but also improves saturation. With this method, however, saturation is unintentionally shifted by the CE process. Even when the saturation improvement process is not conducted, the saturation is slightly changed in accordance with CE.

On the basis of our previous method, this paper proposes an extended reversible image processing method. The proposed method can individually improve saturation and enhance contrast by using the HSV cone model. In the HSV cone model, saturation is obtained as an integer value. Therefore, no complex procedures are required to correct saturation distortion due to the CE process. The proposed method is designed to be effectively applied to devices with a limited storage capacity. We can easily recover an original image from an output image without increasing the data amount by embedding recovery information into the body data. Evaluation results are given to show that our method controls saturation and contrast reversibly and independently.

The rest of the paper is organized as follows. Section 2 gives background information for understanding the proposed method, including an overview of related work. In Section 3, our proposed reversible image processing method for color images is described. The experimental results and analysis are provided in Section 4. Finally, we conclude the paper and describe our future work in Section 5.

## 2. Preparation

### 2.1. HSV Color Space

The HSV color space consists of hue (H), saturation (S), and brightness (V). This color space is classified into two types: the cylinder model [19] and cone model [20,21], depending on the saturation formula. The HSV components are generally given by:

(1) $$H=\left\{\begin{array}{cc} 60\times \frac{G-B}{Max-Min} & if\;Max=R\\ 60\times (\frac{B-R}{Max-Min}+2) & if\;Max=G\\ 60\times (\frac{R-G}{Max-Min}+4) & if\;Max=B\\ notdefined & if\;Max=0, \end{array}\right.$$

(2) $$S_{cylinder}=\left\{\begin{array}{cc} \frac{Max-Min}{Max} & if\;Max\neq 0\\ 0 & if\;Max=0, \end{array}\right.$$

(3) $$S_{cone}=Max-Min,$$

(4) V=Max,

where Max, Median, and Min represent the largest, middle, and smallest values in the RGB components of each pixel and $S_{cylinder}$ and $S_{cone}$ indicate the saturation for the cylinder and cone models, respectively. Note that all variables are used as scalar values. The previous method [13] refers to the cylinder model. The cylinder model has saturation with a fractional value with Equation (2). Therefore, rounding errors are caused in saturation control with the previous method. In contrast, the proposed method uses the cone model. Since the cone model has saturation with an integer value with Equation (3), our method can reversibly improve saturation without any errors. Our proposed method executes three main processes, i.e., saturation improvement, CE, and hue preservation.

### 2.2. Contrast Enhancement Method for Color Images

**Figure 1:**
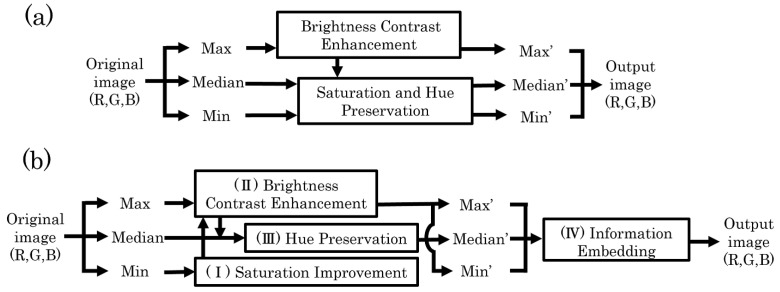
Block diagrams of previous methods. (**a**) Previous method [13]; (**b**) previous method [15].

Wu et al. proposed a CE method for color images [13]. Figure 1a shows a block diagram of this method. First, the R, G, and B values of each pixel are divided into Max, Median, and Min, which are determined by the magnitude relation among the R, G, and B values. Then, the HS-based RDH method [1] is performed on Max to enhance the contrast. Finally, Median and Min are adjusted to retain the original hue and saturation. Accordingly, a contrast-enhanced image without distortion can be obtained.

Here, if the enhancement process is conducted in the HSV color space, a number of rounding errors are caused by color space conversion between RGB and HSV. Those errors degrade the quality of restored images. Although this method cannot completely retrieve the original images, restored images must have a high image quality without artifacts. Therefore, this method deals with providing reversibility by indirectly controlling the H, S, and V values with Max, Median, and Min. In what follows, we describe the CE process for brightness and the preservation of saturation and hue.

#### 2.2.1. Contrast Enhancement

The HS-based RDH method [1] is performed on Max to enhance contrast. In this operation, each pixel value *f* is modified to $f^{\prime }$ by:

(5) $$f^{\prime }=\left\{\begin{array}{cc} f-1 & if\;f<f_{L}\\ f-b_{i} & if\;f=f_{L}\\ f & if\;f_{L}<f<f_{R}\\ f+b_{i} & if\;f=f_{R}\\ f+1 & if\;f>f_{R}, \end{array}\right.$$

where $b_{i}$ is the *i*-th payload bit and $f_{L}$, $f_{R}$ are the highest two bins in the histogram ($f_{L}<f_{R}$). Note that the preprocessing [8] is preliminarily conducted to prevent overflow (OF) and underflow (UF). The information essential to restoring the original image should be embedded with the payload.

#### 2.2.2. Preservation of Saturation and Hue

To prevent the shifting of hue and saturation caused by the CE process, Median and Min are adjusted to retain ratios $C_{S}$ and $C_{H}$, respectively,

(6) $$C_{S}=\frac{Min}{Max}≃\frac{Min^{\prime }}{Max^{\prime },}$$

(7) $$C_{H}=\frac{Median-Min}{Max-Min}≃\frac{Median^{\prime }-Min^{\prime }}{Max^{\prime }-Min^{\prime },}$$

where $Max^{\prime }$ is the value after the processing by Equation (5) and $Median^{\prime }$, $Min^{\prime }$ are the values after the adjustment by Equations (6) and (7). If $Median^{\prime }$ and $Min^{\prime }$ have fractional values, a floor function is applied to them, and those values are converted to integer values.

The previous method applies CE to color images. In the recovery process, Max can be recovered completely. Median and Min, however, cannot be retrieved due to errors caused by applying the floor function. Thus, this method does not guarantee full reversibility. To tackle this issue, we previously proposed a fully reversible image processing method [15]. This method not only enhances contrast, but also improves saturation.

### 2.3. Saturation Improvement and Contrast Enhancement Method for Color Images with Perfect Reversibility

The previous method [15] reversibly controls not only contrast, but also saturation. Figure 1b shows a block diagram of this method. This method has been studied on the basis of a previous method [13], but guarantees complete reversibility by embedding additional information for recovery using the PEE-HS method [18]. Further, the method not only enhances the contrast, but also improves the saturation. The enhancement levels for contrast and saturation can be individually controlled by introducing two parameters for them. This method performs the HS method [1] on Max, which corresponds to the brightness, to enhance the contrast. However, as can be seen from Equation (2), the saturation should be shifted by controlling Max. Nevertheless, this method does not consider adjusting Min to maintain the saturation. Thus, the saturation changes appreciably in accordance with the CE process, even when saturation improvement is not intended. To solve this issue, we propose an extended method of [15] in the next section. The proposed method uses the HSV cone model to prevent unintended changes in saturation and achieve perfectly independent control of contrast and saturation.

## 3. Proposed Method

We propose a reversible image processing method for color images that can improve saturation and enhance contrast independently. The method guarantees reversibility, and the original images can be fully recovered in any case. Each level of saturation improvement and CE can be controlled by using two distinct parameters. We describe the improvement and enhancement process and the recovery process as follows.

### 3.1. Saturation Improvement and Contrast Enhancement Process

**Figure 2:**
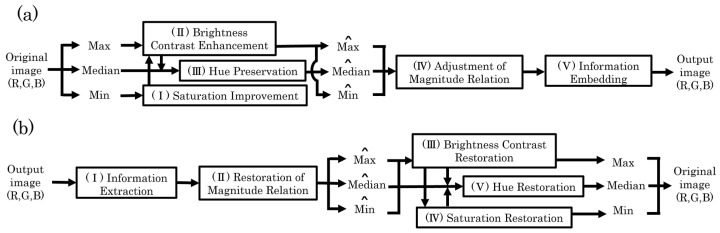
Block diagrams of the proposed method. (**a**) Saturation improvement and contrast enhancement process; (**b**) recovery process.

Figure 2a shows a block diagram of the proposed method. In our method, the R, G, and B values of each pixel are divided into Max, Median, and Min. Saturation is first improved by decreasing the values of Min. The histograms of Max and Min are merged, and then, the preprocessing [8] is conducted to prevent OF and UF. The merged histogram is separated into Max and Min histograms again. The CE process is performed for Max by the HS-based RDH method [1]. In synchronization with the CE process, Min is adjusted to prevent saturation distortion caused by the CE process. Median is subsequently updated to maintain the hue of the original image. $\hat{Max}$, $\hat{Median}$, and $\hat{Min}$ are further calibrated to maintain the magnitude relation among RGB components. They are turned back to R, G, and B values, respectively. Finally, additional information, which is required to recover the original image, is embedded into each color component by using the PEE-HS method [18]. Accordingly, the proposed method can independently improve saturation and enhance contrast with full reversibility. We separately explain each process in detail.

#### 3.1.1. Saturation Improvement

The proposed method improves saturation by decreasing the values of Min on the basis of Equation (3). The procedure consists of two steps.

**Figure 3:**
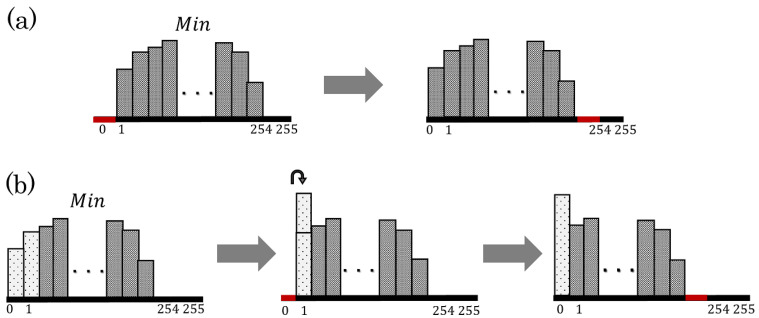
Histogram transition in Min for saturation improvement. (**a**) In case the reference bin is empty (Step 2 (Case 1)); (**b**) in case the reference bin is not empty (Step 2 (Case 2)).

**Step** **1:** Define the leftmost bin of the histogram as the reference bin. In the case that the number of pixels contained in the reference bin is more than 1% of the total number of pixels, the right adjacent bin is defined as the alternative reference bin;
**Step** **2:** [Case 1] In the case that the reference bin is empty, shift the histogram between the reference and the rightmost bins by −1 (see Figure 3a);
  [Case 2] Otherwise, move all the pixels in the reference bin to the right adjacent bin, and shift the histogram between the reference and the rightmost bins by −1 (see Figure 3b).

The threshold of 1% in Step 1 is a desirable parameter determined from our experiments. The above steps are repeated $I_{S}$ times. The improvement level can be controlled by the number of repetitions $I_{S}$. Note that the essential information for recovering original images is embedded in Section 3.1.5.

#### 3.1.2. Contrast Enhancement

**Figure 4:**
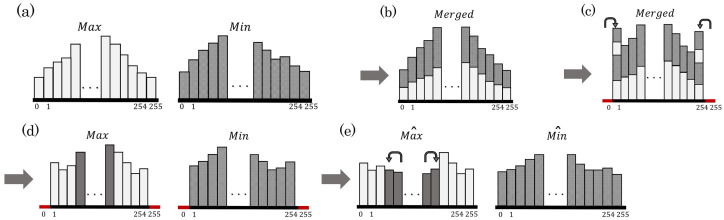
Histogram transition of Min and Max for CE. (**a**) Original histograms of Min and Max; (**b**) merged histogram; (**c**) merged histogram after preprocessing [8]; (**d**) histograms separated into Min and Max; (**e**) Max histogram after CE process and Min histogram after adjustment.

The HS-based RDH method [1] is performed on Max to enhance contrast. Figure 4 illustrates an example of the CE process.

**Step** **1:** Merge histograms of Max and Min (see Figure 4b);
**Step** **2:** Conduct preprocessing [8] to prevent OF and UF (see Figure 4c);
**Step** **3:** Separate the merged histogram into Max and Min histograms (see Figure 4d);
**Step** **4:** Perform the HS-based RDH method [1] on Max to enhance the contrast, and obtain $\hat{Max}$ (see Figure 4e);
**Step** **5:** To prevent saturation distortion, extend Equation (3) to
  (8) $$S_{cone}=\hat{Max}-\hat{Min},$$
  and obtain the $\hat{Min}$ that satisfies Equation (8) (see Figure 4e).

The above steps are repeated $I_{V}$ times. Our method can control the enhancement level by the number of repetitions $I_{V}$. The essential information for restoring the original image is embedded in Step 4. If the information amount exceeds the hiding capacity, the surplus will be embedded in Section 3.1.5.

#### 3.1.3. Hue Preservation

Both saturation improvement and CE practically cause hue distortion. Therefore, the proposed method needs to adjust Median to preserve the original hue. Here, we extend Equation (7) to:

(9) $$C_{H}=\frac{Median-Min}{Max-Min}≃\frac{\hat{Median}-\hat{Min}}{\hat{Max}-\hat{Min}.}$$

According to Equation (9), Median is updated to $\hat{Median}$ so as not to cause hue distortion. If $\hat{Median}$ has a fractional number, the proposed method applies a floor function to $\hat{Median}$. This rounding process causes errors in Median and prevents the original image from being restored. Therefore, the errors should be stored as additional information. We give details on such information in Section 3.1.5.

#### 3.1.4. Adjustment for Magnitude Relation

**Figure 5:**
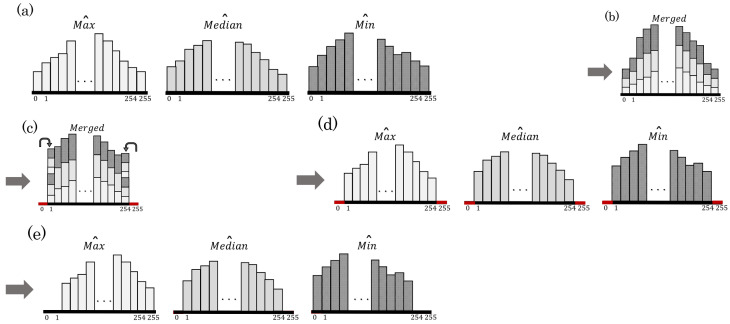
Histogram transition of $\hat{Max}$, $\hat{Median}$, and $\hat{Min}$ in the adjustment for magnitude relation. (**a**) Histograms of $\hat{Max}$, $\hat{Median}$, and $\hat{Min}$; (**b**) merged histogram; (**c**) merged histogram after preprocessing [8]; (**d**) histograms separated into $\hat{Max}$, $\hat{Median}$ and $\hat{Min}$; (**e**) histograms of $\hat{Max}$ shifted by +1, $\hat{Min}$ shifted by −1, and $\hat{Median}$.

After the hue preservation described in Section 3.1.3, the magnitude relation should be $\hat{Max}>\hat{Median}>\hat{Min}$. In some cases, however, the magnitude relation might turn out to be $\hat{Max}\geq \hat{Median}\geq \hat{Min}$. Since the R, G, and B values in each pixel are divided into $\hat{Max}$, $\hat{Median}$, and $\hat{Min}$ again in the recovery process, the proposed method needs to adjust each component to ensure the relation of $\hat{Max}>\hat{Median}>\hat{Min}$, as shown in Figure 5. First, histograms of $\hat{Max}$, $\hat{Median}$, and $\hat{Min}$ are merged (see Figure 5b). Then, both edge bins are emptied using the previous method [8] (see Figure 5c). The merged histogram is separated into three components (see Figure 5d). Finally, the histograms of $\hat{Max}$ and $\hat{Min}$ are shifted by +1 and −1, respectively (see Figure 5e). Accordingly, the proposed method ensures the magnitude relation of $\hat{Max}>\hat{Median}>\hat{Min}$. Note that the essential information for recovering the former relation is embedded in Section 3.1.5.

#### 3.1.5. Guarantee of Reversibility

Additional information is embedded to recover original images in the final process of our method. We used the PEE-HS method [18] for embedding the information, but any arbitrary RDH method can be used. In the proposed method, we need to store four types of additional information to restore the original images. The first and second types are the original bin data, which are lost by the saturation improvement process in Section 3.1.1, and the recovery data, which are required in the recovery process for HS in Section 3.1.2. The third type is rounding errors between Median and $Median^{\prime }$ in Section 3.1.3. Finally, the original bin data of both ends of the histogram, which is lost by adjusting the magnitude relation among Max, Median, and Min, is also required in the recovery process of Section 3.1.4. The first, third, and fourth types of additional information are embedded using PEE-HS. In contrast, the HS process in Section 3.1.2 is an RDH technique; some information can be embedded into an image in this process. Thus, the second type of information, that is the recovery data in Section 3.1.2, is embedded during the HS process up to its maximum capacity. In the case that the data amount is larger than the hiding capacity, the surplus would be embedded here with other additional information. In what follows, we describe the two kinds of additional information in Section 3.1.1 and Section 3.1.3 to restore saturation and hue, respectively:

(i) Additional Information in Section 3.1.1.
  We need to restore the original bin data, which are lost during the saturation improvement process described in Section 3.1.1. The original bin data consists of three types of main data. One of them is an 8 bit pixel value of the reference bin in Step 1. Another is 1 bit classification data of the separate cases (Case 1 or 2) in Step 2. Finally, in the case of Case 2 in Step 2, another piece of 1 bit data is required for each merged pixel to discriminate the pixels in the reference bin from the pixels in the adjacent bin; both bins are merged into a single bin in Case 2 of Step 2. The above data are required in every single process. When the saturation improvement process is repeated $I_{S}$ times, $I_{S}$ sets of data should be stored by the proposed method;
(ii) Additional Information in Section 3.1.3.
  The proposed method applies a floor function to $\hat{Median}$ if $\hat{Median}$ has a fractional number by Equation (9). This rounding process causes errors in Median and prevents the original image from being restored. Therefore, the errors should be stored as additional information. A location map is first derived to record pixels with rounding errors. Then, the map and each error value are compressed by the JBIG2 standard [22] and Huffman coding, respectively.

Finally, all of the additional information is embedded into RGB components by using the PEE-HS method [18]. To reduce the amount of additional information, we adopted the preprocessing from another method [8] as that of the PEE-HS method.

### 3.2. Recovery Process

We describe the recovery process for reverting an output image to the original in accordance with Figure 2b. First, the additional information is extracted from each color component by using the PEE-HS method [18]. The R, G, and B values of each pixel are divided into $\hat{Max}$, $\hat{Median}$, and $\hat{Min}$. The original $\hat{Max}$, $\hat{Median}$, and $\hat{Min}$, which are the values before the process in Section 3.1.4, are restored. Then, the contrast of $\hat{Max}$ is turned back to the original by the HS-based RDH method [1] and Max is fully recovered. The original saturation is subsequently restored, and Min is perfectly reconstructed. The hue is recovered by Equation (9), and the rounding errors in Median are modified simultaneously. Accordingly, the original Median is obtained. Max, Median, and Min are finally turned back to R, G, and B values, respectively. In this way, the proposed method can completely recover the original image.

## 4. Experimental Results

We evaluated the output images derived by the proposed method and three previous methods [1,13,15] in terms of brightness, saturation, and reversibility. We used 24 color images with a size of 768 × 512 pixels downloaded from the Kodak Lossless True Color Image Suite [23] and six color images with a size of 512 × 512 pixels downloaded from the USC website [24]. If we were to use larger-sized images, we could expect that the maximum levels of saturation improvement and CE would be larger. This is because the data hiding capacity should increase. In contrast, if we were to use smaller-sized images, their maximum levels would be smaller. Note that saturation is derived by Equation (3) on the basis of the HSV cone model.

### 4.1. Control of Saturation Improvement and Contrast Enhancement

**Figure 6:**
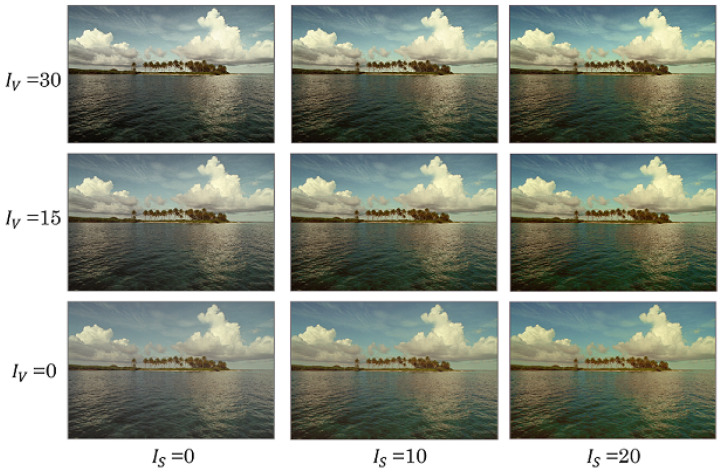
Images output by proposed method, where $I_{S}=0,10,20$ and $I_{V}=0,15,30$ (kodim16).

**Figure 7:**
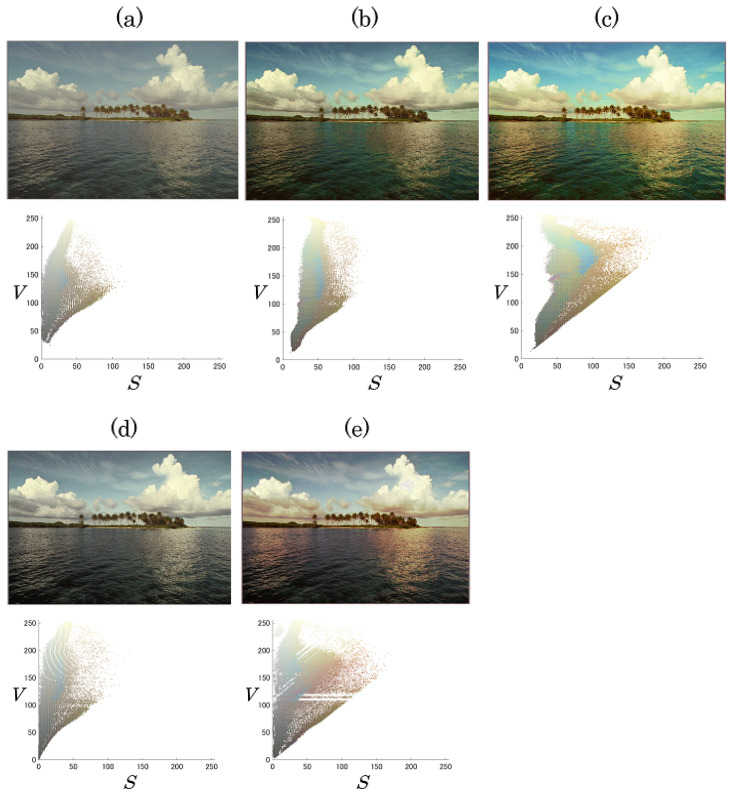
Original and output images and their pixel distributions for saturation and brightness, where $I_{S}$ = 20 and $I_{V}$ = 30 (kodim16). (**a**) Original image; (**b**) proposed method; (**c**) previous method [15]; (**d**) previous method [13]; (**e**) previous method [1].

**Figure 8:**
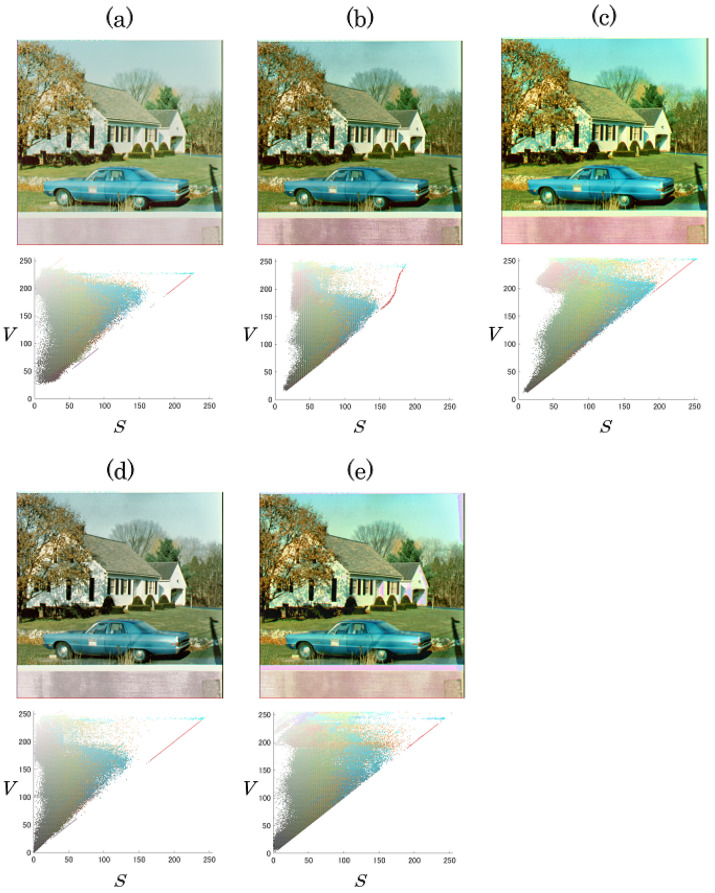
Original and output images and their pixel distributions for saturation and brightness, where $I_{S}$ = 20 and $I_{V}$ = 30 (house). (**a**) Original image; (**b**) proposed method; (**c**) previous method [15]; (**d**) previous method [13]; (**e**) previous method [1].

We first evaluated the output images in terms of saturation improvement, CE, and hue distortion. Figure 6 shows output images derived by the proposed method with $I_{S}=0,10,20$ and $I_{V}=0,15,30$. The proposed method could independently control the levels of saturation improvement and CE by using the individual parameters $I_{S}$ and $I_{V}$. In contrast, the previous methods [1,13] perform CE, but saturation improvement is not considered. Figure 7 and Figure 8 exhibit the original image, images output by each method, and their pixel distributions for the saturation (*S*) and brightness (*V*), where $I_{S}=20$ and $I_{V}=30$. Figure 7b,c and Figure 8b,c reveal that the proposed method and the previous method [15] not only enhanced the contrast, but also improved the saturation. In terms of saturation enhancement, the previous method [15] outperformed the proposed method. This is because it shifts the saturation along with CE. In other words, unintended changes in saturation are caused by CE in this method. As shown in Figure 7d and Figure 8d, the previous method [13] could enhance contrast without hue distortion; this method, however, does not consider saturation improvement. Additionally, from Figure 7e and Figure 8e, it is obvious that CE leads to hue distortion in the previous method [1].

Next, we compared the effects of saturation improvement and CE for the proposed and the previous methods. Table 1 and Table 2 show the results obtained for four different indexes. These tables show the mean values of each evaluation index for all the test images. The improvement level for saturation was assessed by the saturation difference. Relative contrast error (RCE) [25] was adopted to evaluate the enhancement level for contrast:

(10) $$RCE=0.5+\frac{std_{V^{\prime }}-std_{V}}{255},$$

where $std_{V}$ and $std_{V^{\prime }}$ represent the standard deviations of the brightness for the original and output images, respectively. RCE ranges from 0–1. When the contrast is enhanced from the original image, the RCE value exceeds 0.5. We subsequently assessed the brightness difference between original and output images using the relative mean brightness error (RMBE) [25].

(11) $$RMBE=1-\frac{\left|mean_{V^{\prime }}-mean_{V}\right|}{255},$$

where $mean_{V}$ and $mean_{V^{\prime }}$ represent the mean values of the brightness for the original and output images, respectively. If the brightness is perfectly preserved, the RMBE should be one. In contrast, when there are any changes in brightness in the output image, the RMBE is less than one.

Further, we confirmed the hue distortion caused by CE and saturation improvement. The absolute difference in hue was calculated for each image. From Table 1 and Table 2, it is clear that the proposed method and the previous method [15] could improve saturation, while the previous methods [1,13] do not consider saturation improvement. In regard to brightness, each method enhanced the contrast with RCE > 0.5. However, CE causes unintended saturation improvement, even when $I_{s}=0$ in the previous method [15]. Therefore, this method cannot perfectly control saturation and contrast independently.

Additionally, from the values of RMBE, we found that each method can approximately maintain the brightness. With respect to the hue, the proposed method and the previous methods [13,15] could preserve the hue. In contrast, the previous method [1] caused serious hue distortion due to the CE process.

Although the proposed method outperformed the previous method, there was still a restriction. To ensure reversibility, we embedded additional information into images after Step 4 in Figure 2a. This embedding process slightly deteriorates the effects of the CE and hue preservation. As the values of $I_{S}$ and $I_{V}$ become larger, these effects further decrease due to the increase in additional information.

### 4.2. Maximum Improvement/Enhancement Levels

**Figure 9:**
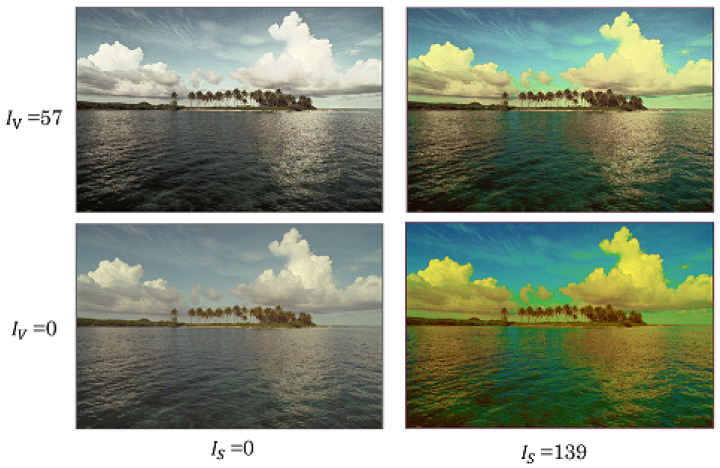
Maximum levels of saturation improvement and CE for proposed method (kodim16).

Figure 9 shows an example of the maximum levels of saturation improvement and CE for kodim16. In this case, $I_{S}=139$ and $I_{V}=57$ are the maximum parameter values. The proposed method flexibly controls the saturation and contrast by changing the parameters within the range of zero to their maximum values.

**Figure 10:**
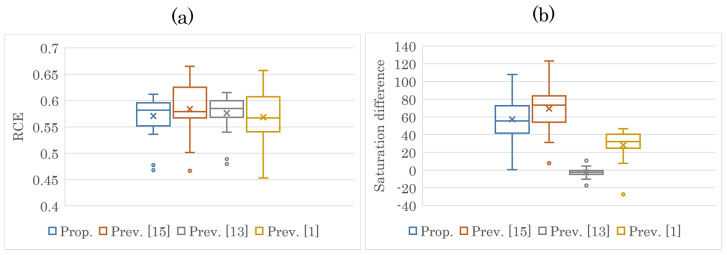
Maximum values of RCE and saturation difference (Kodak). (**a**) RCE; (**b**) saturation difference.

**Figure 11:**
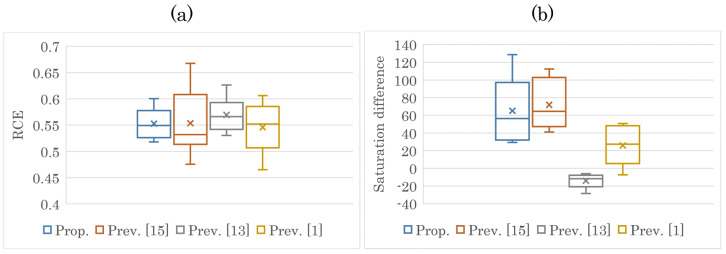
Maximum values of RCE and saturation difference (SIPI). (**a**) RCE; (**b**) saturation difference.

We further analyzed the maximum levels of saturation improvement and CE. Figure 10 and Figure 11 show the maximum values of saturation difference and RCE for all the images output by each method. In regard to brightness, Figure 10a and Figure 11a prove that each method greatly enhanced the contrast. As can be seen from Figure 10b and Figure 11b, the saturation improvement effect was achieved only with the proposed method and the previous method [15].

### 4.3. Reversibility

In the previous sections, we claimed that the original images could be completely reconstructed by our method and the previous methods [1,15], while the previous method [13] could not restore original images. To confirm the reversibility of our method and previous methods [1,13,15], we evaluated the quality of the restored images by using the PSNR, SSIM [26], and CIEDE2000 [27]. With respect to SSIM, we used MSSIM, which is the mean value of SSIM across all windows. In regard to CIEDE2000, as the color difference between the original and output images becomes larger, CIEDE2000 shows a larger value. If there is no color difference, the value should be zero. We conducted the experiment with $I_{V}=30$ and $I_{S}=20$. Table 3 shows the PSNR, MSSIM, and CIEDE2000 values of the restored images obtained by each method. It is shown that all the test images could be perfectly recovered with the proposed method and previous methods [1,15]. Contrarily, although the restored images had high quality with the method [13], their originals were never retrieved.

## 5. Conclusions

We proposed an efficient reversible image processing method for color images that not only enhances contrast, but also improves saturation. The proposed method tackles the issues of our previous work, and it attains fully independent control of both saturation and contrast by adopting the HSV cone model. Each saturation value is obtained as an integer in the HSV cone model. Therefore, we can simply correct the saturation distortion caused by the CE process. The experimental results demonstrated the full reversibility and independent control of saturation and contrast with the proposed method. Our future work involves studying reversibility for other image processing techniques such as smoothing and sharpening. In particular, we will embed information on rounding errors into output images to guarantee reversibility, and we will investigate changing the operator coefficients for flexible control under the process of reversing.

## Figures and Tables

**Table 1:** Effects of saturation improvement and CE (Kodak).

		Saturation		Brightness				Hue	
		Difference		RCE		RMBE		Absolute Difference (Degree)	
		$I_{V}$ = 15	$I_{V}$ = 30	$I_{V}$ = 15	$I_{V}$ = 30	$I_{V}$ = 15	$I_{V}$ = 30	$I_{V}$ = 15	$I_{V}$ = 30
Proposed	$I_{S}$ = 0	0.4029	−3.0155	0.5323	0.5563	0.9851	0.9756	2.6876	3.9587
	$I_{S}$ = 20	16.7591	11.3774	0.5291	0.5525	0.9826	0.9748	1.4316	1.7219
Previous [15]	$I_{S}$ = 0	7.2804	13.7035	0.5299	0.5593	0.9497	0.9251	1.7418	2.1487
	$I_{S}$ = 20	27.8330	35.9077	0.5229	0.5453	0.9297	0.8843	1.2443	1.2685
Previous [13]		−0.4791	−0.9375	0.5331	0.5591	0.9845	0.9762	0.8756	1.0093
Previous [1]		1.4789	4.4302	0.5313	0.5544	0.9827	0.9661	16.7952	30.9996

**Table 2:** Effects of saturation improvement and CE (SIPI).

		Saturation		Brightness				Hue	
		Difference		RCE		RMBE		Absolute Difference (Degree)	
		$I_{V}$ = 15	$I_{V}$ = 30	$I_{V}$ = 15	$I_{V}$ = 30	$I_{V}$ = 15	$I_{V}$ = 30	$I_{V}$ = 15	$I_{V}$ = 30
Proposed	$I_{S}$ = 0	−0.5919	−8.1052	0.5231	0.5402	0.9762	0.9494	2.3316	3.8966
	$I_{S}$=20	14.5529	4.9184	0.5207	0.5354	0.9788	0.9521	1.4352	2.1973
Previous [15]	$I_{S}$ = 0	5.9738	7.5418	0.5252	0.5386	0.9738	0.9682	1.4148	1.7000
	$I_{S}$ = 20	26.0265	25.0130	0.5190	0.5262	0.9481	0.9654	1.0613	1.1762
Previous [13]		−3.9957	−8.4343	0.5257	0.5475	0.9710	0.9370	0.3849	0.5881
Previous [1]		−0.0133	1.9117	0.5294	0.5552	0.9852	0.9703	12.3555	26.4007

**Table 3:** Restored image quality.

	Kodak Image Database			USC Image Database		
	PSNR (dB)	MSSIM	CIEDE2000	PSNR (dB)	MSSIM	CIEDE2000
Proposed	Inf.	1.0000	0.0000	Inf.	1.0000	0.0000
Previous [15]	Inf.	1.0000	0.0000	Inf.	1.0000	0.0000
Previous [13]	59.9088	0.9998	0.1181	58.3516	0.9999	0.1015
Previous [1]	Inf.	1.0000	0.0000	Inf.	1.0000	0.0000

## Data Availability

The data presented in this study are available upon request from the corresponding author.
